# Suitability of Semolina, Cracked Wheat and Cracked Maize as Feeding Commodities for *Tribolium castaneum* (Herbst; Coleoptera: Tenebrionidae)

**DOI:** 10.3390/insects11020099

**Published:** 2020-02-02

**Authors:** Anna Skourti, Nickolas G. Kavallieratos, Nikos E. Papanikolaou

**Affiliations:** 1 Laboratory of Agricultural Zoology and Entomology, Department of Crop Science, Agricultural University of Athens, 75 Iera Odos str., 11855 Athens, Attica, Greece; annaskourti@aua.gr (A.S.); nick_kaval@aua.gr (N.G.K.); 2 Directorate of Plant Produce Protection, Greek Ministry of Rural Development and Food, 150 Sygrou Ave., 17671 Athens, Attica, Greece

**Keywords:** red flour beetle, amylaceous commodities, development, survival analysis

## Abstract

In the current study it was investigated the suitability of semolina, cracked wheat and cracked maize as feeding commodities for the red flour beetle, *Tribolium castaneum* (Herbst) (Coleoptera: Tenebrionidae). The pest completed its development on all tested commodities. The developmental time of larvae was lower on cracked wheat (59.6 days) and cracked maize (54.6 days) compared to semolina (72.8 days). The developmental time of pupae did not differ significantly among tested commodities, ranging from 6.2 to 6.6 days. Female and male longevities were 70.9 and 77.1 days, 92.2 and 77.9 days and 177.0 and 183.7 days, when *T. castaneum* was fed on semolina, cracked wheat and cracked maize, respectively. The highest fecundity (28.7 eggs/female) was recorded when *T. castaneum* was fed on semolina, followed by cracked wheat (2.7 eggs/female) and cracked maize (1.2 eggs/female). The prolonged adult longevity, which was observed on cracked maize, may be attributed to the absence of the cost of reproduction, due to low fecundity on this commodity. The values of the intrinsic rate of increase were 0.014 and −0.021 females/female/day when it was fed on semolina and cracked wheat, respectively, while no demographic analysis was carried out for cracked maize due to high early larval mortality and low fecundity on this commodity. The net reproductive rate and mean generation time were 6.19 females/female and 127.5 days and 0.16 females/female and 91.9 days, when it was fed on semolina and cracked wheat, respectively. Based on demographic analysis, *T. castaneum* population growth is favored only on semolina. We expect semolina to act as a suitable commodity for *T. castaneum*, while cracked wheat and cracked maize allow only its survival by acting as alternative commodities. The estimated demographic parameters of *T. castaneum* on the tested commodities could be used as a useful tool to predict its population outcome in storage facilities.

## 1. Introduction

The quality and quantity of stored products are often associated with arthropod pests [1,2]. Several insects, mainly belonging to Coleoptera and Lepidoptera, are known as serious pests of stored products [3]. The red flour beetle, *Tribolium castaneum* (Herbst; Coleoptera: Tenebrionidae), albeit of Indo-Australian origin, is a worldwide-dispersed stored-product insect pest of high economic importance [4,5,6,7]. Although it causes extensive damage to oilseeds, nuts, spices, dried fruits, pulses, coffee or cocoa, it principally prefers cereals and their related products [8,9,10]. As a secondary pest, *T. castaneum* commonly feeds upon damaged grain kernels, with preference to embryos of grains and flours [7,11,12]. This species is abundant in flour mills and its presence varies according the location inside mills and the type of the facility [13,14]. *Tribolium castaneum* is also able to initiate infestation on grains even before their harvest. For example, Giles [15] identified field infestation of sorghum by several stored-product Coleoptera, including *T. castaneum*, in Northern Nigeria.

The issue of the effective management of insect pests in storage facilities is often faced by experts of applied entomology. Knowledge of the biology of these pests is important, since it provides useful information about their functionality and therefore contributes to the decision-making concerning their management [12,16]. Development, survival and reproduction, which are affected by the consumed food [17,18], are fundamental biological parameters that allow the evaluation of pest performance in different habitats [16,19,20]. The nutritional value of the insect diet can be assessed by the aforementioned parameters, as well as by insect population growth [17]. For instance, the survival of immature stages of insects, the female fecundity and the population growth can be negatively affected by food of low nutritional value [16].

Demography contributes to the understanding of the potential population growth of living organisms [20]. By studying insects’ cohorts from birth to death, several useful conclusions can be deduced for their biology through the calculation of various population parameters and the construction of life tables [16,20,21,22,23,24,25]. For example, the knowledge of the intrinsic rate of increase and net reproductive rate are indicators of its future population increase or decrease, a fact that has various practical applications (e.g., prediction of population level at a given time and comparison between different species) [26].

Although some biological features of *T. castaneum* such as development, reproduction and mortality under varying biotic and abiotic conditions have been previously studied, e.g., [5,12,27,28,29,30,31], there is a lack of literature on the effect of various commodities on the population level by calculating its demographic parameters. For instance, Li and Arbogast [32] examined how the condition (cracked vs. no cracked) of stored-maize affects the demography of *T. castaneum*. A recent exploration concerning the demography of *T. castaneum* infesting ten varieties of barley revealed that two of them were unsuitable for the pest’s population growth [33]. Therefore, the aim of this study was to fill out the existing knowledge of the biology of *T. castaneum* by studying the suitability of semolina, cracked wheat and cracked maize as feeding commodities for this important stored-product insect. Our approach is illustrated via the comparative study of the development, survival and reproduction of *T. castaneum* on these commodities, as well as on its life table statistics. 

## 2. Materials and Methods

### 2.1. Insects

The colonies of *T. castaneum* were reared on white soft wheat flour (variety mixture, made from the endosperm only) at 30 °C, 65% relative humidity and continuous darkness. The founding individuals of *T. castaneum* were collected from a Greek (southern Greece) storage facility in 2003. The culture was kept at the Laboratory of Agricultural Zoology and Entomology, Agricultural University of Athens.

### 2.2. Commodities

Semolina (hard wheat variety mixture), cracked wheat (var. Claudio) and cracked maize (var. Dias) that were clean, free of infestation and pesticides were used in the experiments. Before the initiation of the experiments, the moisture of the tested commodities was adjusted at 13.5% by heating them in an oven at 50 °C [34,35]. The moisture was tested with a calibrated moisture meter (mini GAC plus, Dickey-John Europe S.A.S., Colombes, France).

### 2.3. Experimental Set-Up

Wheat and maize were cracked in a hand-mill. Then, each broken commodity was sieved with two different sieves: a No 8 (2.36 mm openings) U.S. standard testing sieve (Advantech Manufacturing, Inc., New Berlin, WI, USA) and a No 10 (2.00 mm openings) U.S. standard testing sieve (Retsch GmbH, Haan, Germany). The grains that remained on the sieve No 10 were used in the tests. Assays were conducted into petri dishes (5.5 cm diameter, 1 cm height). The internal vertical surface of each dish was covered by polytetrafluoroethylene (60 wt % dispersion in water) (Sigma-Aldrich Chemie GmbH, Taufkirchen, Germany) to prevent individuals that would attempt to escape. The lid of each dish had a central circular opening (1.50 cm diameter) that was covered with muslin gauze to permit adequate aeration in the internal space of dishes. Then, the dishes were separately filled with 5 g of a certain commodity. All quantities of 5 g were weighed with a Precisa XB3200D compact balance (Alpha Analytical Instruments, Gerakas, Greece). To collect eggs of *T. castaneum*, 50 unsexed adults, approximately 7 days old, were transferred from the culture to a 250 mL glass container filled with 125 mL pre-sieved white soft flour for 24 h at 30 °C, 65% relative humidity and continuous darkness. The day after, adults were removed from the flour with a No 20 U.S. standard testing sieve (Advantech Manufacturing, Inc., New Berlin, WI), while eggs were separated from the flour with a No 60 U.S. standard testing sieve (Advantech Manufacturing, Inc., New Berlin, WI) since they remained on the mesh openings of the sieve. The eggs were placed very carefully into dishes separately with a fine brush (Cotman 111 No 000, Winsor and Newton, London, UK) without flour. Dishes were placed in an incubator set at 30 °C, 65% relative humidity and continuous darkness and were inspected daily under an SZX9 Olympus stereomicroscope (57× total magnification; Bacacos S.A., Athens, Greece) for larval emergence. Totally 143, 161 and 146 eggs were used to obtain egg-to-adult development on semolina, cracked wheat and cracked maize respectively. The newly emerged *T. castaneum* larvae were very carefully transferred with a fine brush (Cotman 111 No 000, Winsor and Newton, London, UK) and were separately placed at each one of the dishes that were previously prepared as described above. Different brushes were used for each commodity. Later, dishes containing larvae were placed in an incubator set at 30 °C, 65% relative humidity and continuous darkness. The dishes were preserved at these conditions for the whole experimental period. Duration and survival of larvae and pupae were estimated daily. Larvae that did not make any move or were dehydrated or their color turned to brown were noted as dead. When insects reached the adult stage, pairs were formed and kept separately in the petri dishes. Adult longevity was observed every 24 h. Female fecundity was also examined by calculating the number of eggs laid per female per day. The sex of *T. castaneum* was determined at the adult stage following the description of Halstead [36].

### 2.4. Statistical Analyses

In order to determine the effect of semolina, cracked wheat and cracked maize on larval and pupal developmental time, adult longevity and female fecundity, data were submitted to Kruskal–Wallis analysis of variance on ranks (Dunn’s test at *α* = 0.05), as the Shapiro–Wilk normality test indicated deviation from a normal distribution. The Kaplan–Meier method was used to estimate the survival curves of *T. castaneum* at each of the examined commodities. Furthermore, the Kaplan–Meier estimate was used to obtain mean survival times and their 95% confidence intervals (C.I.). These analyses were conducted using SigmaPlot [37].

Using data on development, survival, fecundity and longevity of *T. castaneum*, we calculated: the net reproductive rate $R_{0}=\sum \left(l_{x}\times m_{x}\right)$, i.e., the per capita rate of offspring production in a period of time equal to cohort study period (*l_x_* corresponds to the cohort survival to age *x* and *m_x_* the age specific fecundity); the intrinsic rate of increase (*r_m_*) $\sum \left(e^{r_{m}\times x}\times l_{x}\times m_{x}\right)=1$, i.e., the rate of natural increase in a closed population (that is subjected to constant age-specific schedules of fertility and mortality for a long period); the finite rate of increase $\lambda =e^{r_{m}}$, i.e., the rate at which the population will increase in each time step and the mean generation time $T=\frac{\ln R_{0}}{r_{m}}$, i.e., the time required for the population to increase by a factor equal to the net reproductive rate. Significant differences between these parameters at each of the examined commodity were tested with the superposition of 95% CIs, which were obtained by bootstrapping in R [38].

## 3. Results

The tested commodities affected the biological features of *T. castaneum* (Table 1). Larval development was significantly longer on semolina (72.8 days) compared to cracked wheat (59.6 days) and cracked maize (54.6 days). However, pupal development was not affected by commodities, ranging from 6.2 to 6.6 days. Female and male longevities were 70.9 and 77.1 days, 92.2 and 77.9 days and 177.0 and 183.7 days, when *T. castaneum* was fed on semolina, cracked wheat and cracked maize, respectively. The highest fecundity (28.7 eggs/female) was recorded when *T. castaneum* was fed on semolina, compared to cracked wheat (2.7 eggs/female) and cracked maize (1.2 eggs/female).

**Figure 1:**
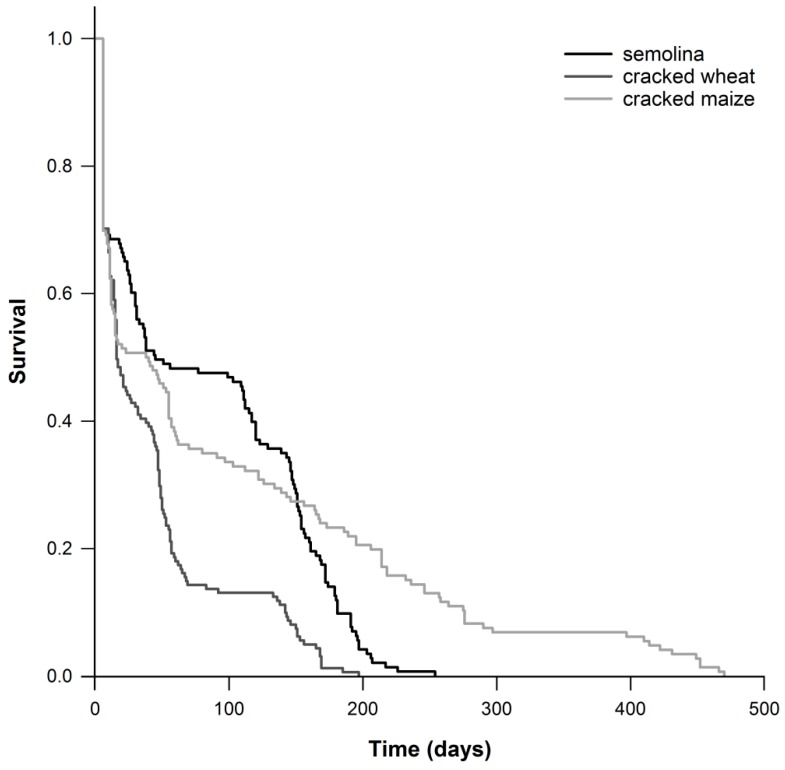
Survival curves of *Tribolium castaneum* fed on three commodities.

The tested commodities also affected the risk of death of *T. castaneum* (*x*^2^ (Log rank) = 41.633; DF = 2; *p* < 0.001; Figure 1). Moreover, *T. castaneum* mean survival time was lower on cracked wheat (41.8 days) compared to semolina (83.9 days) and cracked maize (97.6 days; Table 2). 

When *T. castaneum* was fed on cracked wheat, no sufficient data were collected in order to conduct demographic analysis, due to the detected high early mortality (Figure 1) and low fecundity (Table 1). The net reproductive value, intrinsic rate of increase, the finite rate of increase and the mean generation time of *T. castaneum* on semolina was 6.19 females/female, 0.014 females/female/day, 1.014 and 127.5 days, respectively (Table 3). In contrast, the value of the intrinsic rate of increase was negative on cracked wheat (−0.021 females/female/day) and the values of the net reproductive rate and the finite rate of increase were lower to 1 (0.16 females/female and 0.979, respectively).

## 4. Discussion

Our study revealed several clear findings regarding the biology of *T. castaneum* when fed on semolina, cracked wheat and cracked maize. This pest completed its development and produced eggs on all these commodities. However, based on the values of the intrinsic and finite rate of increase, as well as the reproductive value, *T. castaneum* was able to increase its population only on semolina. This is notably important, as semolina is a common stored-product commodity [39]. Therefore, we expected to degrade both qualitatively and quantitatively this commodity, since it can be characterized as suitable for this pest. The recorded values of the intrinsic and finite rate of increase were lower than the corresponding ones of other stored-product insects, indicating that *T. castaneum* had a lower population increase potential even on semolina. For example, the khapra beetle, *Trogoderma granarium* Everts (Coleoptera: Dermestidae) exhibited 2.50–4.21, 2.79–5.51 or 3.36–4.71 times higher values of intrinsic rate of increase and 1.02–1.05, 1.03–1.07 or 1.04–1.05 when fed on different varieties of barley, hybrids of maize or grain commodities, respectively [16,40,41]. Rahimi Namin et al. [33] reported shorter durations of development of larvae, shorter longevities of female or male adults and higher values of the intrinsic or finite rate of increase when *T. castaneum* fed on different varieties of barley than the observed corresponding values of the current study on semolina. Although the experiments by Rahimi Namin et al. [33] were conducted under the same temperature as in our study, relative humidity was higher (75% ± 5%). Howe [42] reported that relative humidity values > 70% accelerate the egg-to-adult development of *T. castaneum* on sieved grinded wheatfeed. Therefore, stored-product insects that belong to the same or different species present different levels of suitability on certain commodities under varying abiotic conditions. 

On the other hand, *T. castaneum* population decreases when fed on cracked wheat and cracked maize. LeCato and Flaherty [27] found that the supplementation of cracked maize with eggs or adults of the Indian meal moth, *Plodia interpunctella* (Hübner; Lepidoptera: Pyralidae) increased the development and offspring production of *T. castaneum*. Cracked maize does not come under the favorable food preferences of *T. castaneum*, an issue that led to the predation of lepidopterous eggs or adults as a dietary supplementation. On the basis of our findings, the developmental time of *T. castaneum* larvae was significantly prolonged when fed on semolina, a more suitable commodity, in comparison to the less suitable commodities of cracked wheat and cracked maize. In contrast, the duration of development of *T. granarium* larvae was significantly lower when fed on the cracked barley, even though it is a suitable commodity, than on cracked oats and cracked triticale, which are less suitable commodities [16]. Therefore, the interval that insects spend as larvae feeding on suitable commodities may be a species and food dependent phenomenon. The low performance of *T. castaneum* when fed on cracked wheat and maize could be attributed in the presence of α-amylase inhibitors since they negatively impact insects’ fecundity, survival and population growth [32,43,44]. In contrast, Li and Arbogast [32] reported that *T. castneum* is able to, successfully though variably, develop under different cracked maize categories. The fact that the authors used a different type of maize (hybrid “Pioneer 3320”) from the one used in our tests could be a reason for the poor development of *T. castaneum* observed here. For instance, Rahimi Namin et al. [33] reported that the time of development and the % survival of *T. castaneum* larvae or pupae, the longevity of adult females and males, and the fecundity varied significantly among ten varieties of barley. As far as it concerns cracked wheat, former studies have documented that progeny production of *T. castaneum* was lower than wheat flour and wheat bran [45] or barley and oats [46]. Ðukić et al. [45] noticed that the particle size (< 0.425 mm) of cracked wheat that was tested was larger than those of wheat bran and flour. We agree with these results since we used cracked wheat with particles sizes that were > 2.00 and < 2.36 mm. In our study, the value of the intrinsic rate of increase is negative when *T. castaneum* was fed on cracked wheat, which means that its population decreases. Cracked wheat and cracked maize varieties tested here may act as alternative nourishment for *T. castaneum*, favoring its survival when no suitable food exists in the storage facility. Previous reports have documented that several types of commodities act as vehicles where stored-product insects stay alive but in very low numbers till preferred commodities become available [34,35,47]. Further experimentation is needed to clarify this issue.

Pupal development did not differ among the tested commodities, indicating that pupae remain unaffected by insect’s diet. This fact has also been observed for *T. granarium* [16]. However, Rahimi Namin et al. [33] found that there were significant differences in the development of *T. castaneum* female and male pupae among various varieties of barley. Given that differences were not constant for both sexes among the tested varieties it may be concluded that the lack of differences in our study and in the one of Kavallieratos et al. [16] is due to the fact that the overall population of female and male pupae was analyzed.

Among the tested commodities, the lowest longevity of *T. castaneum* adults was on semolina and the highest on cracked maize. This fact may be a result of the cost of the reproduction concept, which refers to increased reproductive activity that may adversely affect the future survival of an organism or its future offspring production [48,49]. Taking into account that the highest fecundity of *T. castaneum* has been reported on semolina, we hypothesized that the reproductive activity of *T. castaneum* on this commodity shortened the adult longevity. On the other hand, the low fecundity of *T. castaneum* on cracked maize resulted in a prolonged adult longevity on this commodity. In contrast, although *T. castaneum* fecundity was also low on cracked wheat, the adult longevity was not prolonged on this commodity. This fact may be an effect of the low nutritional value of cracked wheat and its negative effect on the life history of *T. castaneum*.

Diet selection is crucial for insects, as for all living organisms [50,51]. Consumers, including insects, maximize their long-term average energy intake rate by optimizing the ratio of their food metabolizable energy content to the required consumption time [52], i.e., their aim is to gain as less time as possible in handling their food when they select their diet. In the case of *T. castaneum*, this ratio has got a higher value on semolina than on cracked wheat and cracked maize. Moreover, the amount of the indigestible matter may affect the foraging behavior of *T. castaneum*, leading it to leave out food of lower nutritional value from its diet [51]. Therefore, cracked wheat and cracked maize may present low nutritional value for *T. castaneum*, although these commodities favor its survival. In addition, non-foraging activities, such as mating and egg production, which are beneficial for this pest, may result from a purely digestion process [51] and are indicative of the higher nutritional value of semolina.

## 5. Conclusions

In the light of our findings, the type of commodity and its procession was highly determinative on the overall biological performance of *T. castaneum*. Our demographic approach allowed an efficient evaluation of the suitability of semolina, cracked wheat and cracked maize as feeding commodities for *T. castaneum*, elucidating its fitness components and calculating the corresponding demographic parameters. Further demographic studies are necessary by testing several wild/ laboratory strains of *T. castaneum*, combinations of *T. castaneum* with other stored-product insects, and varieties/hybrids that are currently used in the agricultural practice under different temperature and relative humidity levels in order to shed light on the complex issue of the development of this important stored-product pest. Commodities that enhance the population growth of *T. castaneum* should be very carefully treated at the post-harvest stage to avoid its rapid colonization and further dispersal as well as concomitant food-losses. Similar attention should be paid even to commodities that resist *T. castaneum* infestations since they can be seriously infested by other stored-product insect species.

## Figures and Tables

**Table 1:** Duration of immature development, female and male longevity in days (mean ± SE, median) and female fecundity (eggs/female) of *Tribolium castaneum* fed on three commodities. Medians within a column followed by the same letter are not statistically different (Kruskal–Wallis analysis of variance on ranks, Dunn’s test at *a* = 0.05). Numbers in parentheses correspond to *n* (number of replicates).

Commodity	Larva	Pupa	Female	Male	Fecundity
Semolina	72.8 ± 0.9	6.3 ± 0.1	70.9 ± 4.9	77.1 ± 6.0	28.7 ± 4.7
	71.0 a	6.0 a	69.0 a	78.0 a	22.0 a
	(67)	(67)	(31)	(36)	(31)
Cracked wheat	59.6 ± 4.1	6.2 ± 0.1	92.2 ± 8.4	77.9 ± 5.9	2.7 ± 1.3
	60.0 b	6.0 a	87.5 ab	81.0 ab	1.5 b
	(21)	(21)	(10)	(11)	(10)
Cracked maize	54.6 ± 2.5	6.6 ± 0.3	177.0 ± 23.8	183.7 ± 24.3	1.2 ± 0.4
	52.0 b	6.0 a	161.0 b	187.5 b	0.0 b
	(47)	(47)	(23)	(24)	(23)
*H*	30.884	0.829	18.451	12.591	23.878
DF	2	2	2	2	2
*p*	< 0.001	0.661	< 0.001	0.002	< 0.001

**Table 2:** Survival times in days (mean, 95% C.I.) of *Tribolium castaneum* fed on three commodities. Means followed by the same letter are not statistically different based on the 95% C.I. criterion.

Commodity	Mean	95% C.I.
Semolina	83.9 a	71.6–96.2
Cracked wheat	41.8 b	34.2–49.3
Cracked maize	97.6 a	77.2–118.1

**Table 3:** Values of net reproductive rate (*R*_0_), intrinsic rate of increase (*r_m_*), finite rate of increase (*λ*) and mean generation time (*T*) of *Tribolium castaneum* (mean, 95% C.I.) fed on three commodities. Where dashes exist no demographic parameters were calculated.

Commodity	Net Reproductive Rate (Females/Female) $R_{0}=\sum \left(l_{x}\times m_{x}\right)$		Intrinsic Rate of Increase (Females/Female/Day) $\sum \left(e^{r_{m}\times x}\times l_{x}\times m_{x}\right)=1$		Finite Rate of Increase $\lambda =e^{r_{m}}$		Mean Generation Time (Days) $T=\frac{\ln R_{0}}{r_{m}}$	
	Mean	95% C.I.	Mean	95% C.I.	Mean	95% C.I.	Mean	95% C.I.
Semolina	6.19	4.05–8.68	0.014	0.011–0.017	1.014	1.011–1.017	127.5	125.9–128.9
Cracked wheat	0.16	0.04–0.34	-0.021	-0.033– -0.012	0.979	0.967–0.987	91.9	83.1–105.9
Cracked maize	-	-	-	-	-	-	-	-
